# Establishment and Phytochemical Analysis of a Callus Culture from *Ageratina pichinchensis* (Asteraceae) and Its Anti-Inflammatory Activity

**DOI:** 10.3390/molecules23061258

**Published:** 2018-05-25

**Authors:** Mariana Sánchez-Ramos, Silvia Marquina Bahena, Antonio Romero-Estrada, Antonio Bernabé-Antonio, Francisco Cruz-Sosa, Judith Gonzálesssz-Christen, Juan José Acevedo-Fernández, Irene Perea-Arango, Laura Alvarez

**Affiliations:** 1 Centro de Investigaciones Químicas-IICBA, Universidad Autónoma del Estado de Morelos, Avenida Universidad 1001, Chamilpa, Cuernavaca, Morelos 62209, Mexico; marianasan_06@hotmail.com (M.S.-R.); smarquina@uaem.mx (S.M.B.); are@uaem.mx (A.R.-E.); 2 Centro de Investigación en Biotecnología, Universidad Autónoma del Estado de Morelos, Avenida Universidad 1001, Chamilpa, Cuernavaca, Morelos 62209, Mexico; iperea@uaem.mx; 3 Centro Universitario de Ciencias Exactas e Ingenierías, Departamento de Madera, Universidad de Guadalajara, Celulosa y Papel, Km. 15.5 Carretera Guadalajara-Nogales, Zapopan, Jalisco 45100, Mexico; bernabe_aa@hotmail.com; 4 Departamento de Biotecnología, Universidad Autónoma Metropolitana-Iztapalapa, Av. San Rafael Atlixco 186, Col. Vicentina, Del. Iztapalapa, Ciudad de Mexico 09340, Mexico; cuhp@xanum.uam.mx; 5 Facultad de Farmacia, Universidad Autónoma del Estado de Morelos, Avenida Universidad 1001, Chamilpa, Cuernavaca, Morelos 62209, Mexico; judith.gonzalez@uaem.mx; 6 Facultad de Medicina, Universidad Autónoma del Estado de Morelos, Calle Leñeros s/n, Col. Los Volcanes, Cuernavaca, Morelos 62359, Mexico; juan.acevedo@uaem.mx

**Keywords:** *Ageratina pichinchensis*, dihydrobenzofuran, 3-epilupeol, callus culture, anti-inflammatory

## Abstract

A protocol was established to produce bioactive compounds in a callus culture of *Ageratina pichinchensis* by using 1 mg L^−1^ NAA with 0.1 mg L^−1^ KIN. The phytochemical study of the EtOAc extract obtained from the callus biomass, allowed the isolation and characterization of eleven secondary metabolites, of which dihydrobenzofuran (**5**) and 3-epilupeol (**7**), showed important anti-inflammatory activity. Compound **5** inhibits in vitro the secretion of NO (IC_50_ = 36.96 ± 1.06 μM), IL-6 (IC_50_ = 73.71 ± 3.21 μM), and TNF-α (IC_50_ = 73.20 ± 5.99 μM) in RAW (Murine macrophage cells) 264.7 macrophages, as well as the activation of NF-κB (40% at 150 μM) in RAW-blue macrophages, while compound **7** has been described that inhibit the in vivo TPA-induced ear edema, and the in vitro production of NO, and the PLA2 enzyme activity. In addition, quantitative GC-MS analysis showed that the anti-inflammatory metabolites **5** and **7** were not detected in the wild plant. Overall, our results indicated that *A. pichinchensis* can be used as an alternative biotechnological resource for obtaining anti-inflammatory compounds. This is the first report of the anti-inflammatory activity of compound **5** and its production in a callus culture of *A. pichinchensis*.

## 1. Introduction

*Ageratina pichinchensis (*Kunth), previously denominated *Eupatorium aschembornianum* [1], belongs to the Asteraceae family, and is an endemic herb that grows in the Mexican state of Morelos. This plant is commonly known as “axihuitl” and has been used in traditional Mexican medicine to treat pain, skin infections, wounds, tumors and gastric ulcers [1,2,3].

Pharmacological evaluations showed that *A. pichinchensis* extracts exhibit antimicrobial [1,4,5], antiulcer [6], gastroprotective [7], and anti-inflammatory [8] activities. A number of clinical trials with different extracts and formulations, further demonstrated the effectiveness in the treatment of chronic venous leg ulcers [9], diabetic foot ulcer [10], and wound healing [8,11,12].

Phytochemical investigations established the presence of chromenes and acetophenones [13,14], benzofurans [1], and flavonoids [8] in different extracts obtained from the aerial parts of *A. pichinchensis.* These groups of compounds, mainly chromenes and bezofurans, are of great importance since they possess a wide variety of biological activities such as anticancer, antimicrobial, antiviral, anti-inflammatory, and antioxidant, between others [15,16].

In fact, investigations carried out with *A. pichinchensis* identified the chromene encecalinol [1], together with taraxerol, and (+)-β-eudesmol [14] as the antifungal constituents. Other chromene, encecanescin, was described with anti-ulcer activity [6], 3,5-diprenyl-4-hydroxyacetophenone with gastroprotective activity [7], and the flavonoid 7-*O*-(β-d-glucopyranosyl)-galactin as the most active compound capable of inducing in vitro cellular overgrowth [8].

To date, all phytochemical studies carried out in *A. pichinchensis* have been conducted directly from the wild plant, contributing to the imbalance in the ecosystem. Whereby, plant cell culture offers an alternative to produce bioactive secondary metabolites under a controlled environment, independent of seasonal and geographical conditions. One of the possibilities for producing valuable metabolites under in vitro conditions is based on callus culture. To our knowledge, chromene and benzofuran production by in vitro cultures has not been reported. Furthermore, there is no information related to the production of these metabolites in callus cultures of *A. pichinchensis.*

In the current research, a callus culture protocol from leaf explants of *A. pichinchensis* was established for the first time. In addition, (+)-β-eudesmol acetate (**1**), desmethoxyencecalin (**2**), the mixture of β-sitosterol (**3**) and stigmasterol (**4**), (2*S*,3*R*)-5-acetyl-7,3α-dihydroxy-2β-(1-isopropenyl)-2,3-dihydrobenzofuran (**5**), β-amyrin (**6**), 3-epilupeol (**7**), stigmasterol glucoside (**8**), campesterol (**9**), *n*-hexadecanoic acid (**10**) and hexadecanoic acid, methyl ester (**11**) were identified in the ethyl acetate extract of callus biomass. Thereafter, the anti-inflammatory activity in 12-*O*-tetradecanoylphorbol 13-acetate (TPA) mouse ear edema model, for the ethyl acetate extract, and inhibitory effects on the nitric oxide (NO), IL-6 and Tumor necrosis factor alpha (TNF-α) secretion in Lipopolysaccharide (LPS)-stimulated RAW 264.7 macrophages and Transcription factor (NF-κB) activation in LPS stimulated RAW-blue macrophages for the isolated compound **5** were demonstrated. Needless to mention that we report for the first time the presence of compounds **1**, **2**, **4**, **5**, **6** and **7** in the callus biomass of *A. pichinchensis*.

## 2. Results and Discussion

### 2.1. Callus Production

**Figure 1:**
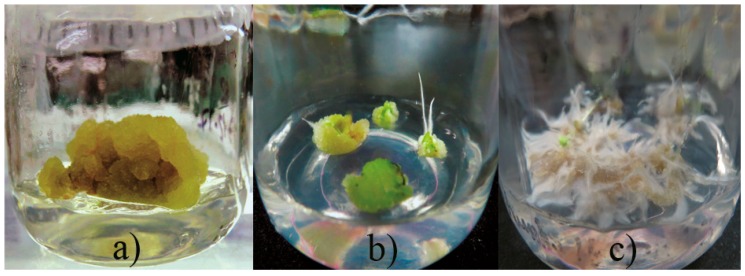
Effect of plant growth regulators on callus induction. (**a**) callus, (**b**) callus with roots, (**c**) roots.

Callus induction was obtained from leaf explants of *A. pichinchensis* seedlings (aged 30-days old) grown under in vitro conditions. Leaf explants were submitted to different concentrations of plant growth regulators (PGR, auxins and cytokinin), such as 2,4-dichlorophenoxyacetic acid (2,4-D), α-naphthaleneacetic acid (NAA), 4-amino-3,5,6-trichloro-2-pyridinecarboxylic acid (picloram) in combination with 6-furfurylaminopurine **(**KIN). It was identified that the best combination of PGR for callus induction and secondary metabolites was NAA and KIN, so a second experimental design was established to identify the appropriate concentration of NAA and KIN (Table 1). Morphogenetic response occurred at 15 days of culture, and greater effect was shown, mainly by combining α-naphthaleneacetic acid (NAA) and kinetin (KIN). The callus produced was friable in appearance and beige in color. The maximum percentage of callus formation (81.7%) was obtained when 1.0 mg L^−1^ NAA with 0.1 mg L^−1^ KIN were added to culture medium (Figure 1a). Simultaneously, callus with roots (20 to 50%, Figure 1b) or roots (15 to 51.7%, Figure 1c) were formed in the treatments containing KIN (1 or 2 mg L^−1^).

This result is like to that obtained for *A. adenophora*, where the highest callus induction frequency (87.2%) was obtained from leaf segments, using MS medium supplemented with 0.5 mg L^−1^ 2,4-dichlorophenoxyacetic acid (2,4-D) and 2.0 mg L^−1^ 6-benzylaminopurine (BA) [17].

### 2.2. Anti-Inflammatory Activity

#### TPA-Induced Auricular Edema in Mice

The ethyl acetate extract obtained from callus biomass of *A. pichinchensis* was evaluated at different doses (0.005 to 0.10 mg/ear) in TPA-induced auricular edema in mice following the protocol described by Gutiérrez-Rebolledo et al. (2016) [18]. This study showed that the extract has an important anti-inflammatory effect. Indeed, the extract reduced the edema in mouse by 35.11 ± 3.79% at the dose of 0.1 mg/ear, which was similar to that obtained for indomethacin (27.66 ± 1.16% inhibition at the dose of 0.1 mg/ear). This result confirms the anti-inflammatory activity of extracts from the wild populations of *A. pichinchensis* previously reported by Romero-Cerecero et al. (2013) [8], who also suggested the presence of secondary metabolites capable to diminishing TPA-induced inflammation. Thus, we continued with the fractionation to identify the active anti-inflammatory compounds.

### 2.3. Chemical Analysis of Callus Biomass

**Figure 2:**
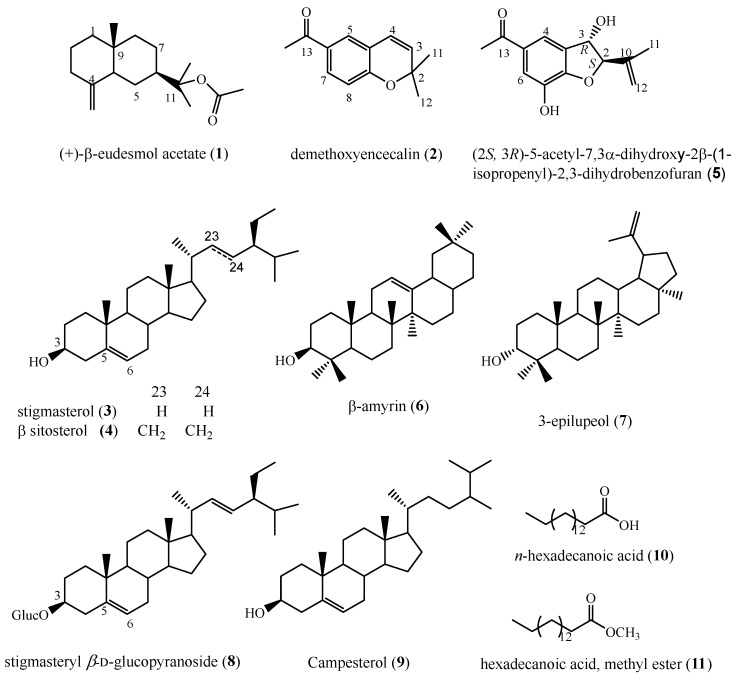
Chemical structures of compounds isolated from callus culture of *A. pichinchensis.*

Repeated silica gel column chromatography of the bioactive extract, allowed the isolation of ten compounds (Figure 2) as described in the Experimental Section. The structures of these compounds were determined as (+)-β-eudesmol acetate (**1**), desmethoxyencecalin (**2**), the mixture of β-sitosterol (**3**) and stigmasterol (**4**), (2*S*,3*R*)-5-acetyl-7,3α-dihydroxy-2β-(1-isopropenyl)-2,3-dihydrobenzofuran (**5**), β-amyrin (**6**), 3-epilupeol (**7**), stigmasterol glucoside (**8**), campesterol (**9**), *n*-hexadecanoic acid (**10**) and hexadecanoic acid, methyl ester (**11**). The structures of compounds **1**–**5** and **8** were determined by comparison of their ^1^H-, ^13^C-NMR, and mass spectrometry data with those reported, while compounds **6**, **9**–**11** were identified by GC-MS (NIST 1.7a). Compounds **3**, **10** and **11** were previously reported as constituents of wild plants of *A. pichinchensis*, while compounds **1**, **2**, **4**–**7** have not been reported from this plant species*.*

Some of the isolated compounds have been described with anti-inflammatory activity; for instance, β-amyrin (**6**) has antinociceptive and anti-inflammatory action (at a dose of 30 mg/Kg) through activation of cannabinoid receptors and by inhibiting cytokine production [19,20,21]. 3-Epilupeol (**7**) inhibit the TPA-induced ear edema, and dose-dependently inhibit NO [22]. Stigmasterol-β-d-glucoside (**8**) and a mixture of β-sitosterol (**3**), stigmasterol (**4**), and campesterol (**9**), isolated from *Budleja globosa*, were able to reduce TPA-induced ear edema [23]. On the other hand, 3-epilupeol (**7**) and hexadecanoic acid (**10**) inhibit the phospholipase A2 enzyme (PLA2) which hydrolyzes phospholipids in the cellular membrane with the consequent release of araquidonic acid (AA), thus initiating the inflammatory response [22,24]. There is no information about the anti-inflammatory activity of the other compounds identified in the callus biomass of *A. pichinchensis*.

### 2.4. In Vitro Anti-Inflammatory Activity

**Figure 3:**
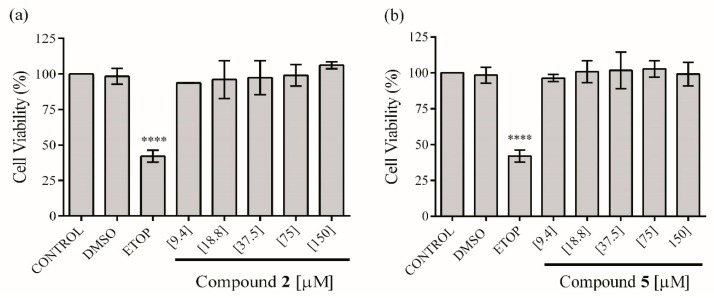
Effect of compounds **2** (**a**) and **5** (**b**) on the viability of RAW 264.7 cells. The values are expressed as the mean ± SD of three independent experiments (*n* = 3). Significance was determined using ANOVA followed by Dunnett’s multiple comparisons test (**** *p* < 0.0001 DMSO, ETOP (etoposide) and compounds compared with control group).

In order to assess the anti-inflammatory activity of the isolated compounds **2** and **5**, these were first evaluated for their cytotoxicity on RAW 264.7 cells. The ethyl acetate extract does not affect the viability of the macrophages up to 30 µg mL^−1^. On the other hand, compounds **2** and **5** at concentrations from 9.4 to 150 μM do not exhibit a significant reduction in viability of macrophages compared with the control group (Figure 3). Therefore, these concentrations were used to test in vitro anti-inflammatory effect of the extract and compounds.

**Figure 4:**
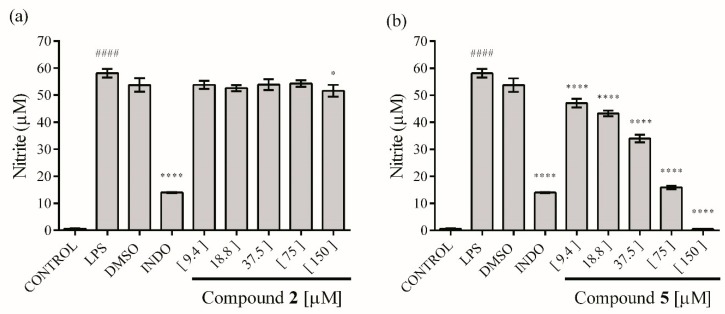
Effect of compounds **2** (**a**) and **5** (**b**) on NO production in lipopolysaccharide (LPS)-stimulated RAW 264.7 macrophages. The values are expressed as the mean ± SD of three independent experiments (*n* = 3). Significance was determined using ANOVA followed by Dunnett’s multiple comparisons test (^####^ *p* < 0.0001 LPS compared with control group; * *p* < 0.05 and **** *p* < 0.0001 DMSO, INDO (indomethacin) and compounds compared with LPS group).

The activity against production of NO and cytokines in macrophages stimulated by LPS was evaluated using RAW 264.7 cells. The results indicated that the ethyl acetate extract showed a limited but significant NO inhibitory activity at 30 µg mL^−1^ (17.52 ± 0.72%). Compound **2** had no significant effect against NO production, even at 150 µM (Figure 4a), nevertheless compound **5** showed an inhibitory effect at all concentrations tested, reaching 100% inhibition at 150 µM, with a dose-dependent relationship (Figure 4b), with a CI_50_ = 36.96 ± 1.06 μM.

**Figure 5:**
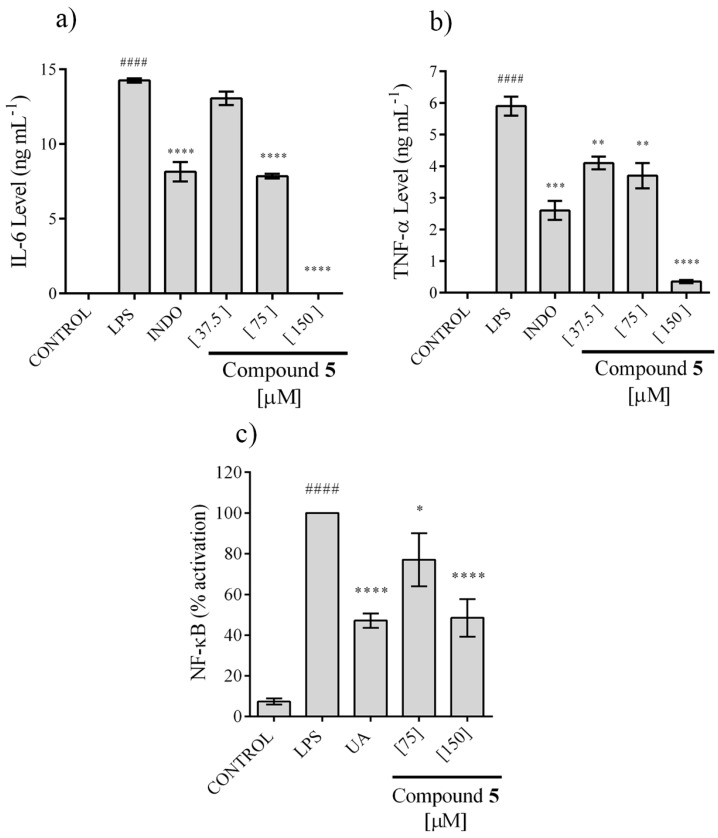
Effect of compound **5** on IL-6 (**a**) and TNF-α (**b**) secretion in LPS-stimulated RAW 264.7, and NF-κB activation in LPS stimulated RAW-blue (**c**). The values are expressed as the mean ± SD of three independent experiments (*n* = 3). Significance was determined using ANOVA followed by Dunnett’s multiple comparisons test (^####^ *p* < 0.0001). LPS compared with control group; * *p* < 0.05, ** *p* < 0.01, *** *p* < 0.001, and **** *p* < 0.0001 INDO (indomethacin) or UA (ursolic acid) or compound **5,** all compared with LPS group.

Because only compound **5** showed activity in the NO assay, it was the only one that was tested for its ability to inhibit Interleukin 6 (IL-6) and TNF-α secretion, two pro-inflammatory cytokines, using the LPS-stimulated RAW cell model. The secretion of IL-6 and TNF-α was totally inhibited at 150 µM and the effect showed a concentration dependence, with a CI_50_ = 73.71 ± 3.21 and 73.20 ± 5.99 μM, respectively (Figure 5a**,**b).

Activation of factor NF-κB is important for the pro-inflammatory response of macrophages, including IL-6 and TNF-α secretion, whereby, the ability of compound **5** to avoid this process was evaluated. We observed that this compound inhibits the activation of NF-κB, but 100% inhibition was not achieved even at 150 µM. Nonetheless, an effect associated with concentration is observed, reaching a 40% of inhibition at 150 µM (Figure 5c).

The NO is an important pro-inflammatory mediator and is involved in some inflammatory disorders including rheumatoid arthritis, chronic hepatitis, and pulmonary fibrosis [25,26]. The results suggested that compound **5** play an important role in the anti-inflammatory activity of *A. pichinchensis*. On the other hand, results indicate that compound **5** could be considered as an anti-inflammation agent because this compound has potential to inhibit NF-κB activation, NO, IL-6 and TNF-α secretion. Overall, our results could suggest that compound **5** suppressed the expression of NF-κB target genes such as Inducible nitric oxide synthase (iNOS) and pro-inflammatory pathway.

### 2.5. Quantification of 2,3-Dihydrobenzofuran (***5***) in Wild Plant and Callus Extracts of A. pichinchensis

These results indicated that compound **5** has an important anti-inflammatory activity, and taken in consideration that 3-epilupeol (**7**) has been described as an anti-inflammatory agent, we decide to quantify both in the callus biomass. For the quantitative GC-MS analyses of **5** and **7** in the EtOAc extracts of wild plant and callus biomass, identification of compounds was obtained by analysis of the peaks at R_T_ = 20.67 min (for **5**) and 38.70 min (for **7**), and the molecular ion peaks at *m*/*z* = 234 for **5** and 426 for **7** observed in the GC-MS analysis (Figures S1 and S2, supplementary material). According to the values of dry biomass (13.92 g), callus from leaf explants produced 0.65 ± 0.011 mg/g dry biomass of compound **5,** and 0.2011 ± 0.015 mg/g dry biomass of 3-epilupeol (**7**) (Figures S3 and S4, supplementary material); by contrast, these compounds were not detected in wild plant extract (Figures S5 and S6, supplementary material). This result indicates that the addition of NAA and KIN (1.0/0.1 mg L^−1^) to the culture medium was essential for production of the anti-inflammatory metabolites **5** and **7** through callus culture derived from segments leaf of in vitro plantlets.

## 3. Materials and Methods

### 3.1. General

Compounds were isolated by means of open column chromatography (CC). Analytical TLC was carried out on precoated silica gel 60F254 plates (Merck, Darmstadt, Germany). The isolation procedures and purity of compounds were checked by thin layer chromatography (TLC), visualized by means of UV light, and sprayed with Ce(SO_4_)_2_ 2(NH_4_)_2_SO_4_ 2H_2_O. All ^1^H- and ^13^C-, DEPT and 2D NMR experiments (COSY, HSQC and HMBC) were recorded on a Bruker AVANCE III HD 500 MHz at 500 and 125 MHz, respectively, using CDCl_3_ with tetramethylsilane (TMS) as internal standard. Mass spectrometry in positive ion mode FABMS was performed using a JEOL JMX-AX505HA mass spectrometer. Optical rotations were measured on a 241 digital polarimeter at 25 °C (Perkin Elmer, Waltham, MA, USA) equipped with a sodium lamp (589 nm) and a microcell. Indomethacin (indo), dimethyl sulfoxide (DMSO), etoposide, lipopolysaccharide (LPS) from *Escherichia coli* serotype 055:B5, sodium nitrite (NaNO_2_), phosphoric acid (H_3_PO_4_), *N*-(1-naphtyl) ethylenediamine dihydrochloride, sulfanilamide and ursolic acid were purchased from Sigma Aldrich (Mexico City, Mexico). ADVANCED DMEM/F12 (Dulbecco’s Modified Eagle Medium/Ham’s F-12), DMEM/F12 (Dulbecco’s Modified Eagle’s Medium/Nutrient Mixture F-12), fetal bovine serum (FBS) and Glutamine (GlutaMax) were from GIBCO (Waltham, MA USA). MycoZap^MT^ Plus-CL antibiotic was from Lonza (Allendale, NJ, USA). [3-(4,5-dimethyl-2-yl)-5-(3-carboxymethoxyphenyl)-2-(4-sulfophenyl)-2*H*-tetrazolium, inner salt; MTS] was from Promega Co (Fitchburg, WI, USA). IL-6 and TNF-α kit was from PEPROTECH (Mexico City, Mexico). QUANTI-Blue^MT^ and Zeocin were from InvivoGen (San Diego, CA, USA). Murine macrophage cell line RAW 264.7 (Tib-71^TM^) was from ATCC^®^ (Georgetown, Washington, DC, USA) and RAW-Blue^TM^ was from InvivoGen. Compounds **5** and **7** were quantified using an HP Agilent Technologies 6890 gas chromatograph equipped with a MSD 5973 quadrupole mass detector (HP Agilent), equipped with a capillary column HP-5MS (length: 30 m; inside diameter: 0.25 mm; film thickness: 0.25 µM). The helium carrier gas was set to the column (1 mL per minute at constant flow). The inlet temperature was set at 250 °C while oven temperature was initially at 40 °C (held for 1 min) and increased to 280 at 10 °C/min. The mass spectrometer was operated in positive electron impact mode with ionization energy of 70 eV. Detection was performed in selective ion-monitoring (SIM) mode and peaks were identified and quantitated using target ions.

### 3.2. Plant Material

Plants and seeds of *Ageratina pichinchensis* were collected in Tepoztlán Morelos, Mexico. Both plant and seed were identified by Biol. Gabriel Flores Franco and deposited at the HUMO Herbarium of the Universidad Autónoma del Estado de Morelos (UAEM), with the voucher number 33913.

### 3.3. Callus Induction and Maintenance

The seeds of *A. pichinchensis* were surface disinfected and germinated in sterile semisolid MS culture medium with 3% sucrose, plant growth regulators (PGRs)-free, pH adjusted to 5.8 and 0.2% phytagel. Culture medium was previously sterilized at 121 °C, 15 psi, for 15 min using an autoclave. After four weeks, the leaves of the seedlings were cut into pieces and four explants were transferred into jars containing 25 mL the same MS medium and PGRs (auxins and cytokinin). First, a screening of tree auxins (α-naphthaleneacetic acid, NAA; 2,4-dichlorophenoxyacetic acid, 2,4-D; picloram, PIC) at 0.1 or 1.0 mg L^−1^, each combined with a cytokinin (kinetin, KIN) at 0.1 mg L^−1^, were evaluated to determine which auxin induced better callus formation at 20 days of culture. At 20 days, we observed that NAA showed better response on leaves explant. Therefore, to determine the best treatment of callus induction, different concentrations of NAA (0.0, 0.1, 1.0 or 2.0 mg L^−1^) were combined with KIN (0.0, 0.1, 1.0 or 2.0 mg L^−1^). Five jars containing four leaf explants for each treatment were used to determine the percentage of callus induction at 20 days of culture and the experiment was repeated three times. Callus subcultures were carried out every 30 days for 12 months using culture medium with the same conditions. All cultures were incubated at 25 ± 2 °C under photoperiod of 16 h with white fluorescent light (50 µmol m^−2^ s^−1^).

### 3.4. Extraction and Isolation

The stable callus was used for the extraction of secondary metabolites. Callus was harvested and dried in an oven at 40 °C. In order to obtain the greatest amount of compound, dry biomass (13.92 g) was extracted three times with 100 mL ethyl acetate by sonication; each extraction was 30 min. The solvent was evaporated under reduced pressure and a syrupy extract (550 mg) was obtained. The ethyl acetate extract was fractionated in an open chromatographic column previously packed with silica gel (15 g, 70–230 mesh; Merck) and eluted with a *n*-hexane/ethyl acetate gradient system (100:00, 95:05, 90:10, 85:15, 80:20, 75:25 and 00:100, *v*/*v*). Fractions of 10 mL were collected to obtain 99 fractions and monitored by TLC (ALUGRAM^®^ SIL G/UV_254_ silica gel plates). Fractions that showed TLC similarity were grouped obtaining 9 groups: MSR 1 (1–13; 81 mg), MSR 2 (14–16; 56 mg), MSR 3 (17–30; 12.3 mg), MSR 4 (31–42; 47 mg), MSR 5 (43–45; 16.5 mg), MSR 6 (46–57; 70.6 mg), MSR 7 (58–68; 62 mg), MSR 8 (69–85; 78.4 mg) and MSR 9 (86–99; 58.9 mg). The less polar fraction MSR 1 showed by TLC a sole compound, and GC-MS analysis indicated that this fraction was constituted by the mixture of *n*-hexadecanoic acid (**10**) and hexadecanoic acid methyl ester (**11**). MSR 2, MSR 4, MSR 6, MSR 7 and MSR 8 fractions obtained from the main column were separately rechromatographed over silica gel using *n*-hexane containing increasing amounts of ethyl acetate (100:00→70:30). *β*-eudesmol acetate (**1**) (5.2 mg; 0.6%) was isolated from MSR 2 fraction; desmethoxyencecalin (**2**) (7.1 mg; 1.3%) from MSR 4 fraction; the mixture of stigmasterol (**3**) and β-sitosterol (**4**) (21.8 mg; 3.9%) was obtained from fraction MSR 6; From fraction MSR 7, 6 mg of 3-epilupeol (**7**) were obtained by crystallization, and GC-MS analysis of the mother liquors allowed the identification of β-amyrin (**6**), and campesterol (**9**). Column chromatographic purification of MSR 8 fraction, using a gradient of *n*-hexane-EtOAc (100:00→70:30) afforded five fractions, fractions 75–80 eluted with 7:3 *n*-hexane-EtOAc contained an oily pure compound characterized as (2*S*,3*R*)-5-acetyl-7,3α-dihydroxy-2β-(1-isopropenyl)-2,3-dihydrobenzofuran (**5**) (12.8 mg, 2.3%). Fractions 81–90, eluted with *n*-hexane-EtOAc (70:30) contained a solid compound identified as stigmasterol-β-d-glucoside (**8**).

Compounds **1**, **2**, **5**, and **7** were identified using ^1^H- and ^13^C-NMR, and comparison with reported values. Compounds **3**, **4** and **8** were characterized by direct comparison with authentic samples available in our laboratory [27,28].

*β-Eudesmol acetate* (**1**). Colorless oil; Rf = 0.87 (*n*-hexane:ethyl acetate; 90:10); ${\left[\alpha \right]}_{D}^{20}$: +16.5° (*c* = 1; CHCl_3_). ^1^H-NMR (200 MHz, CDCl_3_) *δ*_H_: 5.03 (1H, d, *J* = 2 Hz, 15a), 4.83 (1H, d, *J* = 2 Hz, H-15b), 2.3–2.43 (2H, m, CH_2_-3), 2.19 (3H, s, CH_3_ at Ac), 1.92 (1H, t, H-10), 1.84 (1H, m, H-6), 1.24–1.68 (10H, m, CH_2_-1, 2, 5, 7 and 8), 1.26 (6H, s, CH_3_ at C-11), 0.84 (3H, s, CH_3_-14). ^13^C-NMR (50 MHz, CDCl_3_) *δ*_C_: 170.21 (CO at Ac), 151.32 (C-4), 108.18 (C-15), 82.65 (C-11), 49.68 (C-10), 43.52 (C-6), 42.14 (C-1), 40.7 (C-8), 36.9 (C-3), 35.83 (C-9), 26.72 (C-14), 24.89 (C-5), 24.32 (C-12), 24.32 (C-13), 22.42 (C-2), 22.58 (C-7), 21.29 (CH_3_ at Ac). These data match those in the literature [29,30].

*Desmethoxyencecalin* (**2**). Colorless oil; Rf = 0.54 (CH_2_Cl_2_:MeOH; 95:05); ^1^H-NMR (200 MHz, CDCl_3_) *δ*_H_: 7.75 (1H, dd, *J* = 2.2, 8.2 Hz, H-7), 7.62 (1H, d, *J* = 2.4 Hz, H-5), 6.82 (1H, d, *J* = 8.2 Hz, H-8), 6.51 (1H, d, *J* = 10.2 Hz, H-4), 5.65 (1H, d, *J* = 10.2 Hz, H-3), 2.04 (3H, s, CH_3_ of Ac), 1.25 (6H, s, geminal CH_3_ at C-2). ^13^C-NMR (50 MHz, CDCl_3_) *δ*_C_: 196.56 (CO of Ac), 157.38 (C-10), 131.24 (C-7), 130.45 (C-6), 130.26 (C-5), 125.78 (C-5), 126.93 (C-3), 121.74 (C-4), 120.67 (C-9), 116.62 (C-8), 77.76 (C-2), 28.42 (CH_3_ at C-2), 28.42 (CH_3_ at C-2), 26.23 (CH_3_ at Ac). These data match those in the literature [31].

(2*S*,3*R*)-5-Acetyl-7,3α-dihydroxy-2β-(1-isopropenyl)-2,3-dihydrobenzofuran (**5**). Yellowish oil; ${\left[\alpha \right]}_{D}^{20}$: −17° (0.82; CHCl_3_); ^1^H-NMR (500 MHz, CDCl_3_) δ_H_: 7.6 (1H, d, *J* = 1.4 Hz, H-4), 7.51 (1H, d, *J* = 1.4 Hz, H-6), 5.2 (1H, d, *J* = 3.8 Hz, H-3), 5.1 (1H, brs, H-12), 5.03 (1H, d, *J* = 3.8 Hz, H-2), 4.96 (1H, brs, H-12), 2.52 (3H, s, H-14), 1.74 (3H, s, H-11). ^13^C-NMR (100 MHz, CDCl_3_) δ_C_: 151.7 (C-8), 140.9 (C-7), 140.6 (C-10), 132.3 (C-5), 129.3 (C-9), 118.9 (C-4), 117.8 (C-6), 113.5 (C-12), 96.0 (C-2), 76.8 (C-3), 17.5 (C-11). These data match those in the literature [32].

*3-Epilupeol* (**7**)*.* Crystalline solid. ^1^H-NMR (400 MHz, CDCl_3_) δ_H_: 4.58 (1H, d, *J* = 2.5 Hz, 29a); 4.56 (1H, d, *J* = 1.2 Hz, 29b); 3.38 (1H, t, *J* = 2.8 Hz, H-3); 2.38 (1H, td, *J* = 11, 5.9 Hz, H-19); 1.88 (2H, c, H-21); 1.69 (2H, m, H-2); 1.68 (3H, s, H-30); 1.65 (2H, t, H-22); 1.54 (2H, m, H-16); 1.45 (1H, m, H-18); 1.40 (1H, m, H-5); 1.38 (2H, m, H-1, H-15); 1.37 (6H, m, H-7, H-11, H-12,); 1.36 (2H, m, H-6); 1.26 (1H, m, H-13); 1.20 (1H, t, H-9); 1.03 (6H, s, CH_3_-23 y 24); 0.96 (3H, s, CH_3_-25); 0.93 (3H, s, CH_3_-26); 0.84 (3H, s, CH_3_-28); 0.82 (3H, s, CH_3_-27). ^13^C-NMR (100 MHz, CDCl_3_) δ_C_: 151.23 (C-20); 109.51 (C-29); 76.47 (C-3); 50.45 (C-9); 49.25 (C-5); 48.53 (C-18); 48.25 (C-19); 43.25 (C-17); 43.13 (C-14); 41.18 (C-8); 40.24 (C-22); 38.26 (C-13); 37.75 (C-4); 37.53 (C-10); 35.82 (C-16); 34.38 (C-7); 33.48 (C-1); 30.09 (C-21); 28.46 (C-23); 27.62 (C-15); 25.64 (C-2); 25.36 (C-12); 22.36 (C-24); 21.02 (C-11); 19.51 (C-30); 18.51 (C-6); 18.23 (C-28); 16.19 (C-25); 16.14 (C-26) and 14.86 (C-27). These data match those in the literature [33].

### 3.5. GC/MS Quantification of the Anti-Inflammatory Metabolites ***5*** and ***7***

For the quantitative analysis, a series of solutions containing the standard molecule (2*S*,3*R*)-5-acetyl-7,3α-dihydroxy-2β-(1-isopropenyl)-2,3-dihydrobenzofuran (**5**) in known concentrations (2.2, 1.1, 0.55, 0.275, 0.1375, 0.06875 mg mL^−1^) and (0.350, 0.175, 0.0875, 0.04375, 0.02187 mg mL^−1^) for the standard molecule 3-epilupeol (**7**) were prepared in triplicate and analyzed by GC-MS. The linear calibration curves were plotted to quantify the compounds **5** and **7** in the ethyl acetate extract obtained from biomass of callus culture. Each standard solution was analyzed in triplicate to calculate the peak area ratio (y) and relative concentration (x), these data were used to construct the linear calibration curve, which showed acceptable linearity with correlation coefficients *r*^2^ = 0.9926 and *r*^2^ = 0.9997, respectively (Figures S1 and S2 of supplementary material). The results were obtained by analysis in Microsoft Excel 2010.

#### Preparation of Extracts for GC-MS Quantification

Dried leaves of *A. pichinchensis* (15.2 g) were exhaustively extracted with EtOAc (3 × 100 mL) by maceration. After extraction and evaporation of the solvent 1.76 g of residue was obtained. An aliquot (12.5 mg mL^−1^) was diluted with chloroform (1 mL) and analyzed by GC-MS.

After 30 days of culture, the calli were harvested, dried and ground into fine powder. The finely ground samples (13.92 g) were extracted with EtOAc (3 × 100 mL) in a sonication bath for 30 min. The EtOAc extracts were filtered under vacuum through Whatman No. 1 filter paper and dried in vacuum to yield 550 mg of EtOAc extract. An aliquot (11.3 mg) was diluted with chloroform (1 mL) and immediately injected in a GC-MS for the quantification of compounds **5** and **7**. The identification was based on the comparison of retention time and MS data with those of the authentic sample. Quantification was performed by using the calibration curve previously constructed in the same conditions and expressed in terms of mg g^−1^ dry mass. The analysis was done using three replicates per treatment.

### 3.6. Anti-Inflammatory Experiments

#### 3.6.1. TPA-Induced Mouse Ear Edema

Mouse ear edema was evaluated according to the method described previously [22]. All experiments were carried out using five animals per treatment. Adult male CD-1 mice with a body weight of 30 ± 5 g, were used. Experiments were performed according to the Official Mexican Rule: NOM-062-ZOO-1999 Guidelines (Technical Specifications for the Production, Care, and Use of Laboratory Animals) and international ethical guidelines for the care and use of experimental animals. The experimental protocol used was approved by Comité para el Cuidado y Uso de los Animales de Laboratorio (CCUAL) de la Facultad de Medicina (No. 06-2015). Mice were maintained under standard laboratory conditions (Bioterio de la Facultad de Medicina, Universidad Autónoma del Estado de Morelos) at 22 °C ± 3 °C, 70% ± 5% of humidity, 12 h light/dark cycle and food/water ad libitum. A negative control group received acetone as vehicle and indomethacin was used as anti-inflammatory drug as positive control group. Animal ear inflammation was induced with 2.5 μg/ear of TPA dissolved in 20 μL of acetone applied to the internal and external surface of the right ear to cause edema. Sample doses of 0.005, 0.01, 0.05, and 0.1 mg/ear of the EtOAc extract, as well as the anti-inflammatory drug of reference indomethacin (0.005, 0.01 and 0.1 mg/ear) were applied. All the samples of the different treatments were dissolved in (10 μL of acetone) and applied topically on the right ear immediately after TPA application; on the left ear, acetone was applied as vehicle. Four hours after application of the samples of interest as possible anti-inflammatory agents, the animals of each treatment were sacrificed by cervical dislocation. Circular sections of 6 mm in diameter were taken from both: the treated (t) and the non-treated (nt) ears, which were weighed to determine the inflammation. Percentage of inhibition was determined by the formula expressed below:

$$\mathrm{Inhibition}\%=\left(\;\frac{∆w\;\mathrm{control}-\;∆w\;\mathrm{treatment}}{∆w}\right)\times 100$$

where Δ_w_ = w_t_ − w_nt_; being w_t_ the weight of the section of the treated ear and w_nt_ the weight of the section of the non-treated ear.

#### 3.6.2. Cell Culture (RAW 264.7)

RAW 264.7 cells were maintained in Advanced DMEM/F12 medium supplemented with 1% GlutaMax^TM^ and 3.5% FBS, without antibiotics. Cells were cultured at 37 °C in a humidified atmosphere containing 5% CO_2_.

#### 3.6.3. Assay for Cell Viability (RAW 264.7)

RAW 264.7 cells (1 × 10^4^ cell/well in 100 µL of medium) were seeded in a 96-well plate and incubated for 24 h, the cells were treated with various concentrations (9.4–150 μM) of compounds **2** and **5** or vehicle (DMSO, 0.5%, *v*/*v*) or etoposide (68 μM) and incubated for 20 h. Cell viability was determined by MTS assay, using the CellTiter 96 Aqueous Non-Radioactive Cell Proliferation assay (Promega), according to the manufacturer’s instructions. Briefly, 20 μL of MTS was added to each well, and the cells were incubated for another 4 h. The optical density was measured at 490 nm on a microplate reader.

#### 3.6.4. Treatment of Macrophages RAW 264.7 with LPS

RAW 264.7 cells (3 × 10^4^ cells/well in 200 µL of medium) were plated and incubated for 24 h at 37 °C into 96-well plates. After that, the cells were incubated for two hours with the compounds **2** and **5** at various concentrations (9.4–150 μM), vehicle (DMSO, 0.5%, *v*/*v*) or indomethacin (84 μM) and then were incubated with LPS (1 µg mL^−1^) at 37 °C for 20 h as a pro-inflammatory stimulus. Finally, cell-free supernatants were collected and a part was used to NO quantification in fresh and another one was kept at −20 °C until IL-6 and TNF-α quantification.

#### 3.6.5. Determination of NO Concentration

Nitrite, the stable end-product of NO, was measured according to Griess reaction. Briefly, 50 μL of each supernatant were mixed with 100 μL of Griess reagent [50 μL of 1% sulfanilamide and 50 μL of 0.1% *N*-(1-naphtyl) ethylenediamine dihydrochloride in 2.5% phosphoric acid], for 10 min at room temperature. The optical density at 540 nm (OD_540_) was measured with a microplate reader and nitrite concentration in the samples were calculated by comparison with the OD_540_ of a standard curve of NaNO_2_ prepared in fresh culture medium [34].

#### 3.6.6. Measurement of IL-6 and TNF-α

The quantitative measurement of murine IL-6 and TNF-α secretion in the supernatants was performed using the PEPROTECH Murine IL-6 and TNF mini ABTS ELISA kit, according to the instructions provided by manufacturer.

#### 3.6.7. Cells Culture (RAW-Blue)

RAW-Blue cells were maintained in DMEM/F12 medium supplemented with 10% FBS, with 1% MycoZap and 200 µg mL^−1^ Zeocin. Cells were cultured at 37 °C in humidified atmosphere containing 5% CO_2_.

#### 3.6.8. Determination of NF-κB Activation

RAW-Blue cells (3 × 10^4^ cells/well in 100 µL of medium) were plated and incubated into 96-well plates for 24 h at 37 °C. After that, the cells were incubated for two hours with the compound **5** at 75 and 150 μM or DMSO (0.5%, *v*/*v*) or indomethacin (84 μM) or ursolic acid (17.5 µM, positive control) and then were incubated with LPS (1 µg mL^−1^) at 37 °C for 20 h to stimulate NF-κB activation [35,36]. Finally, cell-free supernatants were collected and were used in fresh to determine NF-κB activation, related to alkaline phosphatase secretion using QUANTI-Blue™ (InvivoGen), according to the instructions provided by manufacturer.

#### 3.6.9. Statistical Analysis

The results shown were obtained at least by three independent experiments and are presented as means ± SDs. Statistical analyses were performed by one-way analysis of variance (ANOVA) followed by Dunnett’s multiple comparisons test. The mean comparation test for callus percentages was performed according to Tukey’s method. All statistical analyses were performed using the GraphPad Prism^®^, Version 6.0 software. *p* values <0.05 were considered to indicate statistical significance.

## 4. Conclusions

A protocol has been developed to produce anti -inflammatory metabolites in callus culture of *A. pichinchensis*. NAA and KIN found to be essential for the production of friable callus. Callus from in vitro leaf explant in presence of 1.0 mg L^−1^ NAA with 0.1 mg L^−1^ KIN in MS medium after 30 days of culture produce eleven secondary metabolites (**1**–**11**), of which, metabolites **3**, **4**, **6**, **7**, **9** and **10** were previously described with anti-inflammatory properties. In this work, we demonstrated that the dihydrobenzofuran **5**, was the major constituent of the callus biomass, and effectively inhibited the LPS-induced production of proinflammatory factors, such as NO, IL-6, and TNF-α in RAW 264.7 macrophages, and the activation of NF-kB in RAW-blue macrophages without causing cytotoxicity. A possible mechanism for this effect involves the ability of **5** to activate a signaling cascade, which results in the repression of NF-κB, in LPS-challenged macrophages. Although further investigation is needed to clarify the precise mechanisms by which **5** inhibits NF-κB activation, this natural dihydrobenzofuran, produced by a callus culture of *A. pichinchensis*, may be considered a potential therapeutic agent for the treatment of inflammation-related disease.

Quantitative analysis demonstrated that the most active compounds **5** and **7** were not produced in wild plants. Therefore, callus culture of *A. pichinchensis* can serve as a good source for the obtaining of anti-inflammatory metabolites. This is the first report of the production of metabolites **1**, **2**, **4**–**7** in a callus culture of *A. pichinchensis*, and the first report of the anti-inflammatory activity of compound. 

## Figures and Tables

**Table 1:** Morphogenetic responses of plant growth regulators (PGRs) on *A. pichinchensis* leaf explant at 20 days culture.

PGRs (mg L^−1^)		Callus (%)	Callus with Roots (%)	Roots (%)
NAA	KIN			
0	0	0.00 ± 0.00 ^e^	0.00 ± 0.00 ^c^	0.00 ± 0.00 ^d^
0	0.1	0.00 ± 0.00 ^e^	0.00 ± 0.00 ^c^	0.00 ± 0.00 ^d^
0	1	0.00 ± 0.00 ^e^	28.33 ± 4.19 ^ab^	33.33 ± 4.35 ^abc^
0	2	0.00 ± 0.00 ^e^	21.67 ± 5.03 ^bc^	51.67 ± 10.40 ^a^
0.1	0	0.00 ± 0.00 ^e^	0.00 ± 0.00 ^c^	0.00 ± 0.00 ^d^
0.1	0.1	0.00 ± 0.00 ^e^	20.00 ± 4.45 ^bc^	36.67 ± 7.52 ^abc^
0.1	1	0.00 ± 0.00 ^e^	33.33 ± 6.37 ^ab^	51.67 ± 8.40 ^a^
0.1	2	0.00 ± 0.00 ^e^	31.67 ± 5.23 ^ab^	40.00 ± 6.25 ^ab^
1	0	30.00 ± 8.66 ^d^	0.00 ± 0.00 ^c^	0.00 ± 0.00 ^d^
1	0.1	81.67 ± 7.64 ^a^	7.50 ± 1.15 ^c^	0.00 ± 0.00 ^d^
1	1	41.67 ±7.64 ^bcd^	36.67 ± 4.04 ^ab^	21.66 ± 3.45 ^bcd^
1	2	5.00 ± 0.61 ^e^	50.00 ± 6.35 ^a^	35.16 ± 7.22 ^abc^
2	0	33.33 ± 5.77 ^cd^	0.00 ± 0.00 ^c^	0.00 ± 0.00 ^d^
2	0.1	51.67 ±12.58 ^b^	33.33 ± 3.84 ^ab^	15.00 ± 4.25 ^cd^
2	1	48.33 ± 4.16 ^bc^	20.00 ± 2.54 ^bc^	0.00 ± 0.00 ^d^
2	2	10.00 ± 1.11 ^e^	30.00 ± 3.76 ^ab^	38.33 ± 2.89 ^ab^

These letters mean that the data is statistically significant.

## Supplementary Materials

Figure S1: GC-MS analyses of 5; Figure S2: GC-MS analyses of 7; Figure S3: GC-MS analysis of pure compound 5; Figure S4: GC-MS analysis of pure compound 7; Figure S5: GC-MS analysis of compound 5 in wild plant; Figure S6: GC-MS analysis of compound 7 in wild plant; Figure S7: 1H-NMR spectrum (400 MHz; CDCl3) of compound 1; Figure S8: 13C-NMR spectrum (100 MHz, CDCl3) of compound 1; Figure S9: 1H-NMR spectrum (400 MHz; CDCl3) of compound 2; Figure S10: 13C-NMR spectrum (100 MHz, CDCl3) of compound 2; Figure S11: 1H-NMR spectrum (500 MHz; CDCl3) of compound 5; Figure S12: 13C-NMR spectrum (125 MHz, CDCl3) of compound 5; Figure S13: 1H-NMR spectrum (400 MHz; CDCl3) of compound 7; Figure S14: 13C-NMR spectrum (100 MHz, CDCl3) of compound 7.

## References

[1] Ríos M.Y., Aguilar-Guadarrama B., Navarro V. (2003). Two new benzofuranes from *Eupatorium aschenbornianum* and their antimicrobial activity. Planta Med..

[2] Argueta A., Cano L., Rodarte M. (1994). Atlas de la Medicina Tradicional Mexicana, Tomo 1–3.

[3] Avilés M., Suárez G. (1994). Catálogo de Plantas Medicinales. Jardín Etnobotánico.

[4] Navarro-García V.M., Gonzalez A., Fuentes M., Aviles M., Rios M.Y., Zepeda G., Rojas M.G. (2003). Antifungical activities of nine traditional Mexican medicinal plants. J. Ethnopharmacol..

[5] Torres-Barajas L., Rojas-Vera J., Morales-Méndez A., Rojas-Fermín L., Lucena M., Buitrago A. (2013). Chemical composition and evaluation of antibacterial activity of essential oils of *Ageratina jahnii* and *Ageratina pichinchensis* collected in Mérida, Venezuela. Bol. Latinoam. Caribe Plant. Med. Aromat..

[6] Sánchez-Mendoza M.E., Reyes-Trejo B., Sánchez-Gómez P., Rodriguez-Silverio J., Castillo-Henkel C., Cervantes-Cuevas H., Arrieta J. (2010). Bioassay-guided isolation of an anti-ulcer chromene from *Eupatorium aschenbornianum*: Role of nitric oxide, prostaglandins and sulfydryls. Fitorerapia.

[7] Sánchez-Mendoza M., Rodriguez-Silverio J., Rivero-Cruz J.F., Rocha-González H., Pineda-Farías J., Arrieta J. (2013). Antinociceptive effect and gastroprotective mechanisms of 3,5-diprenyl-4-hydroxyacetophenone from *Ageratina pichinchensis*. Fitoterapia.

[8] Romero-Cerecero O., Zamilpa A., González-Cortazar M., Alonso-Cortés D., Jiménez-Ferrer E., Nicasio-Torres P., Aguilar-Santamaría L., Tortoriello J. (2013). Pharmacological and chemical study to identify wound-healing active compounds in *Ageratina pichinchensis*. Planta Med..

[9] Romero O., Zamilpa A., Jiménez E., Tortoriello J. (2011). Exploratory study on the effectiveness of a standardized extract from of *Ageratina pichinchensis* in patients with Chronic venous leg ulcers. Planta Med..

[10] Romero O., Zamilpa A., Tortoriello J. (2015). Effectiveness and tolerability of standardized extract from *Ageratina pichinchensis* in patients with diabetic food ulcer: A randomized, controlled pilot study. Planta Med..

[11] Romero O., Zamilpa A., Ramos A., Alonso D., Jiménez J., Huerta M., Tortoriello J. (2011). Effect on the wound healing process and in vitro cell proliferation by the medical mexican plant *Ageratina pichinchensis*. Planta Med..

[12] Romero C., Zamilpa A., Díaz G., Tortoriello J. (2014). Pharmacological effect of *Ageratina pichinchensis* on wound healing in diabetic rats and genotoxicity evaluation. J. Etnopharmacol..

[13] Gómez F., Quijano L., Calderón J., Perales A., Ríos T. (1982). 2,2-Dimethylchromenes from *Eupatorium aschembornianum*. Phytochemistry.

[14] Aguilar-Guadarrama B., Navarro V., León-Rivera I., Ríos M.Y. (2009). Active compounds against tinea pedis dermatophytes from *Ageratina pichinchensis* var. bustamenta. Nat. Prod. Res..

[15] Nevagi R., Dighe N., Dighe S. (2015). Biological and medicinal significance of benzofuran. Eur. J. Med. Chem..

[16] Costa M., Dias T., Brito A., Proenca F. (2016). Biological importance of structurally diversified chromenes. Eur. J. Med. Chem..

[17] Shen J., Li X., Wang D., Lu H. (2007). *In vitro* culture of croftonweed (*Ageratina adenophora*): Considerable potential for fast and convenient plantlet production. Weed Technol..

[18] Gutiérrez-Rebolledo G., Garduño-Siciliano L., García-Rodríguez R., Pérez-González M., Chávez M.I., Bah M., Siordia-Reyes G., Chamorro-Cevallos G., Jiménez-Arellano M.A. (2016). Anti-inflammatory and toxicological evaluation of *Moussonia deppeana* (Schldl. & Cham) Hanst and verbascoside as a main active metabolite. J. Ethnopharmacol..

[19] Da Silva K.A., Paszcuk A.F., Passos G.F., Silva E.S., Bento A.F., Meotti F.C., Calixto J.B. (2011). Activation of cannabinoid receptors by the pentacyclic triterpene α,β-amyrin. Pain.

[20] Chicca A., Marazzi J., Gertsch J. (2012). The antinociceptive triterpene β-amyrin inhibits 2-arachidonoylglycerol (2-AG) hydrolysis without directly targeting cannabinoid receptors. Br. J. Pharmacol..

[21] Shih M.F., Cherng J.Y. (2014). Reduction of adhesion molecule production and alteration of eNOS and endothelin-1 mRNA expression in endothelium by *Euphorbia hirta* L. through its beneficial β-amyrin molecule. Molecules.

[22] Romero-Estrada A., Maldonado M.A., González-Christen J., Marquina B.S., Garduño-Ramírez M.L., Rodríguez-López V., Alvarez L. (2016). Anti-inflammatory and antioxidative effects of six pentacyclic triterpenes isolated from the Mexican copal resin of *Bursera copallifera*. BMC Complement. Altern. Med..

[23] Backhouse N., Rosales I., Apablaza C., Goity I., Erazo S., Negrete R., Thedoluz C., Rodríguez J., Delporte C. (2008). Analgesic, anti-inflammatory and antioxidant properties of *Buddleja globosa*, Buddlejaceae. J. Ethnopharmacol..

[24] Aparna V., Dileep K.V., Mandal P.K., Karthe P., Sadasivan C., Haridas M. (2012). Antiinflammatory property of n-hexadecanoic acid: Structural evidence and kinetic assessment. Chem. Biol. Drug Des..

[25] Dong R., Yuan J., Wu S., Huang J., Xu X., Wu Z., Gao H. (2015). Anti-inflammation furanoditerpenoids from *Caesalpinia minax* Hance. Phytochemistry.

[26] Kanwar J.R., Kanwar R.K., Burrow H., Baratchi S. (2009). Recent advances on the roles of NO in cancer and chronic inflammatory disorders. Curr. Med. Chem..

[27] Meckes M., Garduño-Ramírez M.L., Marquina S., Alvarez L. (2001). Iridoides adicionales de la planta medicinal *Astianthus viminalis* y su actividad hipoglucemiante y antihiperglucemiante. Rev. Soc. Quím. Mexico.

[28] Alvarez L., Herrera-Arellano A., Marquina S., Tortoriello J., Zamilpa A., González M., Villarreal M.L., Martínez-Rivera M.A., López-Villegas E.D., Rodríguez-Tovar A.V. (2009). Anti-mycotic and anti-inflammatory constituents from four Mexican medicinal *Solanum* species. Curr. Top. Steroid Res..

[29] Mincione E., Iavarone C. (1972). Terpenes from Arabian *Commiphora myrra*. I. Chim. Ind..

[30] Jena S., Ray A., Banerjee A., Sahoo A., Nasim N., Sahoo S., Kar B., Patnaik J., Panda P., Nayak S. (2017). Chemical composition and antioxidant activity of essential oil from leaves and rhizomes of *Curcuma angustifolia* Roxb. Nat. Prod. Res..

[31] Willuhn G., Junior I., Wendisch D. (1986). Desmethoxyencecalin and thymol derivatives from *Arnica sachalinensis*. Planta Med..

[32] Zhou Z.Y., Liu W.X., Pei G., Ren H., Wang J., Xu Q.L., Xie H.H., Wan F.H., Tan J.W. (2013). Phenolics from *Ageratina adenophora* roots and their phytotoxic effects on *Arabidopsis thaliana* seed germination and seedlind growth. J. Agric. Food Chem..

[33] Torres-Naranjo M., Suarez A., Gilardoni G., Cartuche L., Flores P., Morocho V. (2016). Chemical constituents of *Muehlenbeckia tomnifolia* (Kunth) Meisn (Polygonaceae) and its in vitro α-amilase and α-glucosidase inhibitory activities. Molecules.

[34] Yang G., Lee K., Ham I., Choi H.-Y. (2012). Inhibition of lipopolysaccharide induced nitric oxide and protaglandin E2 production by chloroform fraction of *Cudrania tricuspidata* in RAW 264.7 macrophages. BMC Complement. Altern. Med..

[35] Checker R., Sandur S.K., Sharma D., Patwardhan R.S., Jayakumar S., Kohli V., Sethi G., Aggarwal B.B., Sainis K.B. (2012). Potent anti-inflamatory activity of ursolic acid, a triterpenoid antioxidant, is mediated throgh suppresion of NF-kB, AP-1 and NF-AT. PLoS ONE.

[36] Shishodia S., Majumdar S., Banerjee S., Aggarwal B.B. (2003). Ursolic acid inhibits nuclear factor-kB activation induced by carcinogenic agents through suppression of IƙBα kinase and p65 phosphorylation: Correlation with down-regulation of cyclooxygenase 2, matrix metalloproteinase 9, and cyclin D1. Cancer Res..

