# Supplementary material for: Establishment and Phytochemical Analysis of a Callus Culture from Ageratina pichinchensis (Asteraceae) and Its Anti-Inflammatory Activity
Source: Molecules. 2018 May 25;23(6):1258. doi: 10.3390/molecules23061258 (PMC6099804; doi:10.3390/molecules23061258)
Supplement: Supplementary file 1 [file molecules-23-01258-s001.pdf]

SUPPLEMENTARY MATERIAL

Establishment and Phytochemical Analysis of a Callus Culture from *Ageratina pichinchensis* (Asteraceae) and Its Anti-Inflammatory Activity

Mariana Sánchez-Ramos [1,2], Silvia Marquina Bahena [1], Antonio Romero-Estrada [1], Antonio Bernabé-Antonio [3], Francisco Cruz-Sosa [4], Judith González-Christen [5], Juan José Acevedo-Fernández [6], Irene Perea-Arango [2] and Laura Alvarez [1,*]

1 Centro de Investigaciones Químicas-IICBA, Universidad Autónoma del Estado de Morelos, Avenida Universidad 1001, Chamilpa, Cuernavaca, Morelos 62209, México; marianasan_06@hotmail.com (M.S.-R.); smarquina@uaem.mx (S.M.B.); are@uaem.mx (A.R.-E.)

2 Centro de Investigación en Biotecnología, Universidad Autónoma del Estado de Morelos, Avenida Universidad 1001, Chamilpa, Cuernavaca, Morelos 62209, México; iperea@uaem.mx

3 Centro Universitario de Ciencias Exactas e Ingenierías, Departamento de Madera, Universidad de Guadalajara, Celulosa y Papel, Km. 15.5 Carretera Guadalajara-Nogales, Zapopan, Jalisco 45100, México; bernabe_aa@hotmail.com

4 Departamento de Biotecnología, Universidad Autónoma Metropolitana-Iztapalapa, Av. San Rafael Atlixco 186, Col. Vicentina, Del. Iztapalapa, Ciudad de México 09340, México; cuhp@xanum.uam.mx

5 Facultad de Farmacia, Universidad Autónoma del Estado de Morelos, Avenida Universidad 1001, Chamilpa, Cuernavaca, Morelos 62209, México; judith.gonzalez@uaem.mx

6 Facultad de Medicina, Universidad Autónoma del Estado de Morelos, Calle Leñeros s/n, Col. Los Volcanes, Cuernavaca, Morelos 62359, México; juan.acevedo@uaem.mx

* Correspondence: lalvarez@uaem.mx

Abstract

A protocol was established to produce bioactive compounds in a callus culture of *Ageratina pichinchensis* by using 1 mg L^{-1} NAA with 0.1 mg L^{-1} KIN. The phytochemical study of the EtOAc extract obtained from the callus biomass, allowed the isolation and characterization of eleven secondary metabolites, of which dihydrobenzofuran (**5**) and 3-epilupeol (**7**), showed important anti-inflammatory activity. Compound **5** inhibits *in vitro* the secretion of NO (IC_{50} = 36.96 ± 1.06 μM), IL-6 (IC_{50} = 73.71 ± 3.21 μM), and TNF (IC_{50} = 73.20 ± 5.99 μM) in RAW 264.7 macrophages, as well as the activation of NF-κB (40 % at 150 μM) in RAW-blue macrophages, while compound **7** has been described that inhibit the *in vivo* TPA-induced ear edema, and the in vitro production of NO, and the PLA2 enzyme activity. In addition, quantitative GC-MS analysis showed that the anti-inflammatory metabolites **5** and **7** were not detected in the wild plant. Overall, our results indicated that *A. pichinchensis* can be used as an alternative biotechnological resource for obtaining anti-inflammatory compounds. This is the first report of the anti-inflammatory activity of compound **5** and its production in a callus culture of *A. pichinchensis*.

Keywords: *Ageratina pichinchensis*; dihydrobenzofuran; 3-epilupeol; callus culture; anti-inflammatory.

List of content

Figure S1. GC-MS analyses of **5** in the EtOAc extract of callus ,identification of compounds was obtained by analysis of the peaks at R_T = 20.67 min.

Figure S2. GC-MS analyses of 7 in the EtOAc extract of callus ,identification of compounds was obtained by analysis of the peaks at R_T = 38.70 min.

Figure S3. GC-MS analysis of pure compound **5**.

Figure S4. GC-MS analysis of pure compound **7**.

Figure S5. GC-MS analysis of compound **5** in wild plant.

Figure S6. GC-MS analysis of compound **7** in wild plant.

Figure S7. ^{1}H NMR spectrum (400 MHz; $CDCl_3$) of compound **1**.

Figure S8. ^{13}C NMR spectrum (100 MHz, $CDCl_3$) of compound **1**.

Figure S9. ^{1}H NMR spectrum (400 MHz; CDCl3) of compound **2**.

Figure S10. ^{13}C NMR spectrum (100 MHz, $CDCl_3$) of compound **2**.

Figure S11. ^{1}H NMR spectrum (500 MHz; $CDCl_3$) of compound **5**.

Figure S12. ^{13}C NMR spectrum (125 MHz, $CDCl_3$) of compound **5**.

Figure S13. ^{1}H NMR spectrum (400 MHz; $CDCl_3$) of compound **7**.

Figure S14. ^{13}C NMR spectrum (100 MHz, $CDCl_3$) of compound **7**.

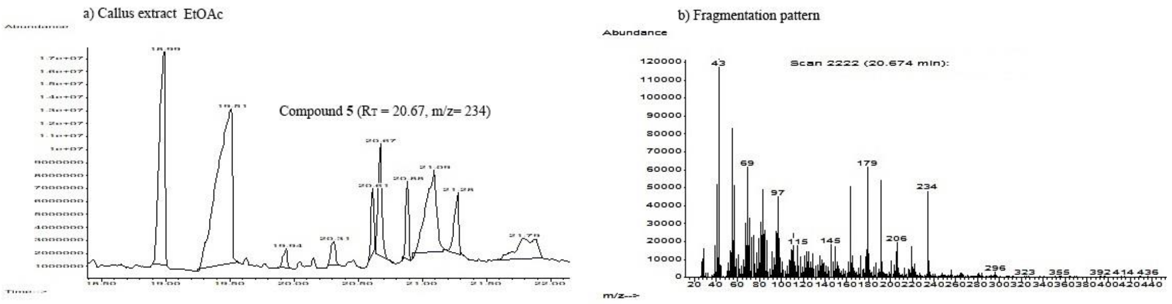

Figure S1. GC-MS analyses of **5** in the EtOAc extract of callus, identification of compound was obtained by analysis of the peak at R_T = 20.67 min.

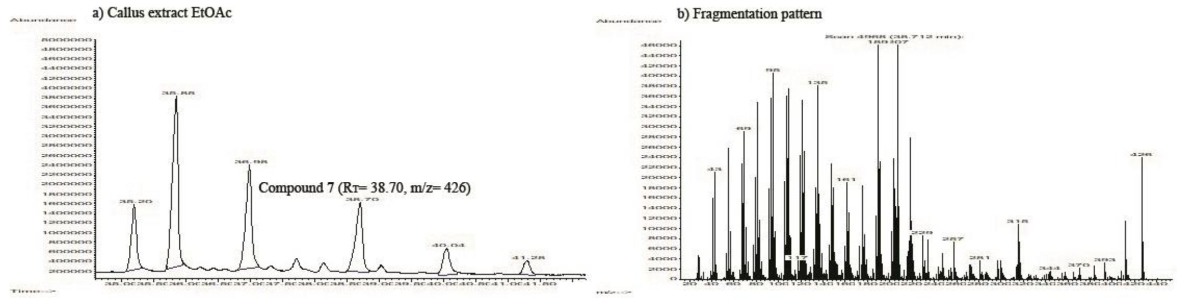

Figure S2. GC-MS analyses of **7** in the EtOAc extract of callus, identification of compound was obtained by analysis of the peak at R_T = 38.7 min.

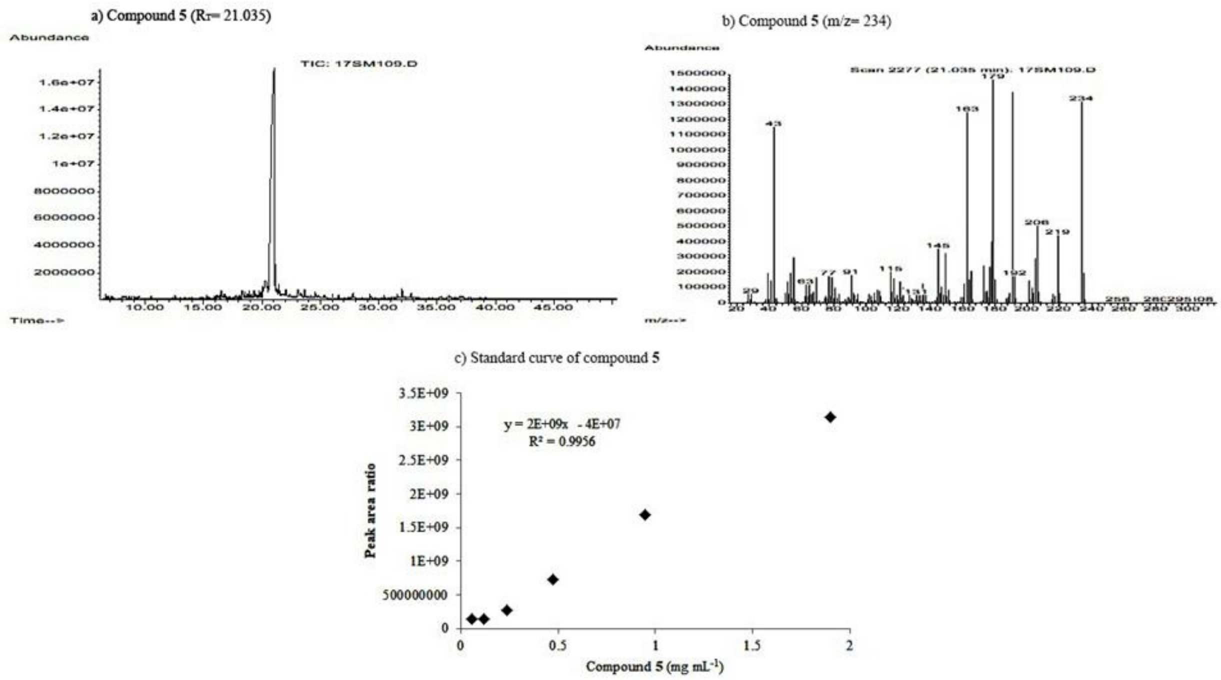

Figure S3. GC-MS analysis of pure compound **5.**

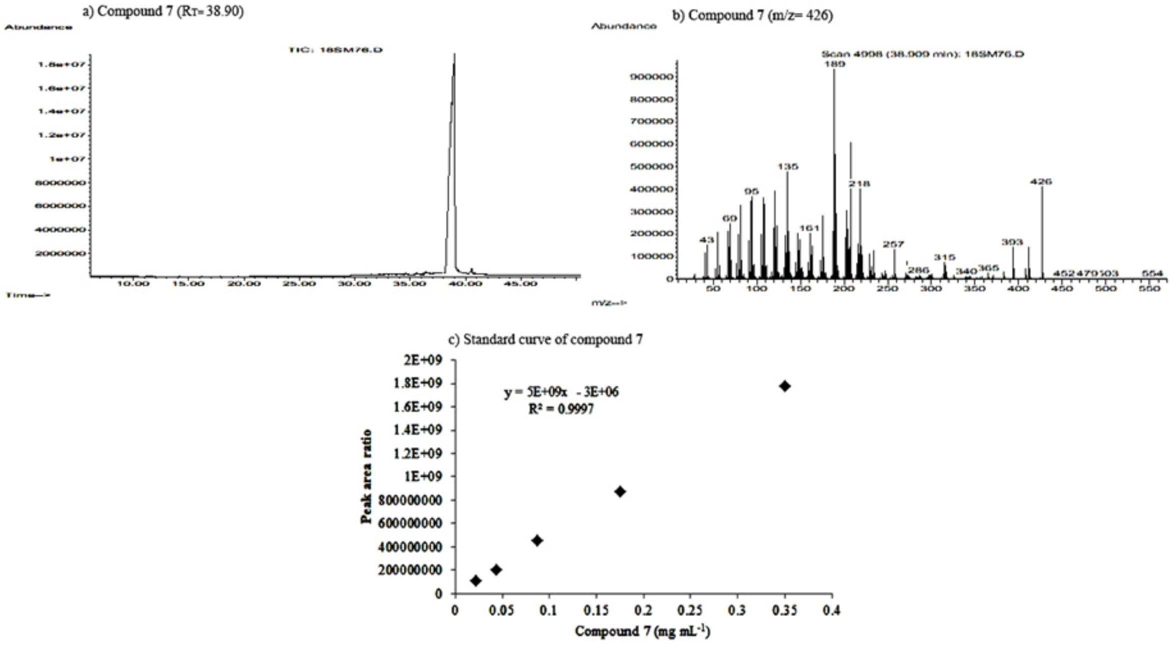

Figure S4. GC-MS analysis of pure compound **7**.

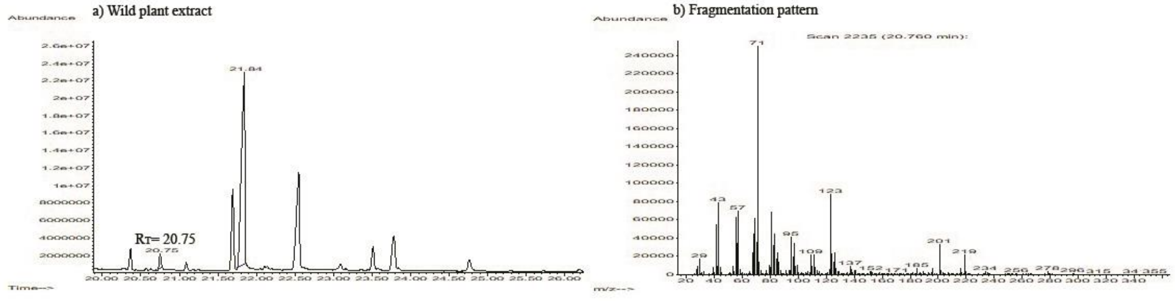

Figure S5. GC-MS analysis of compound **5** in wild plant.

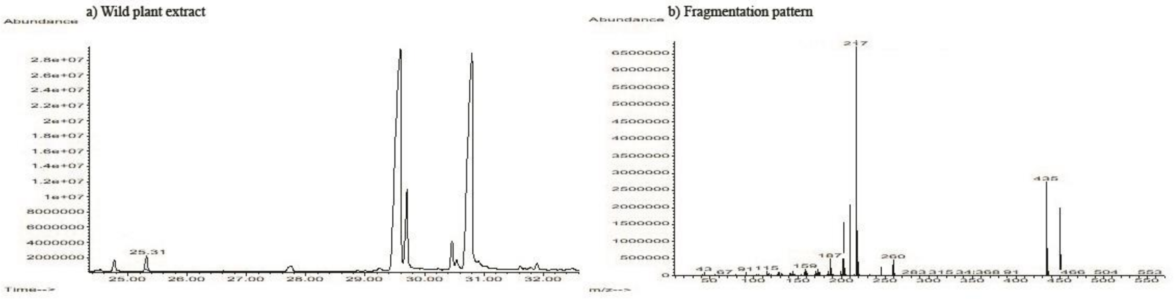

Figure S6. GC-MS analysis of compound **7** in wild plant.

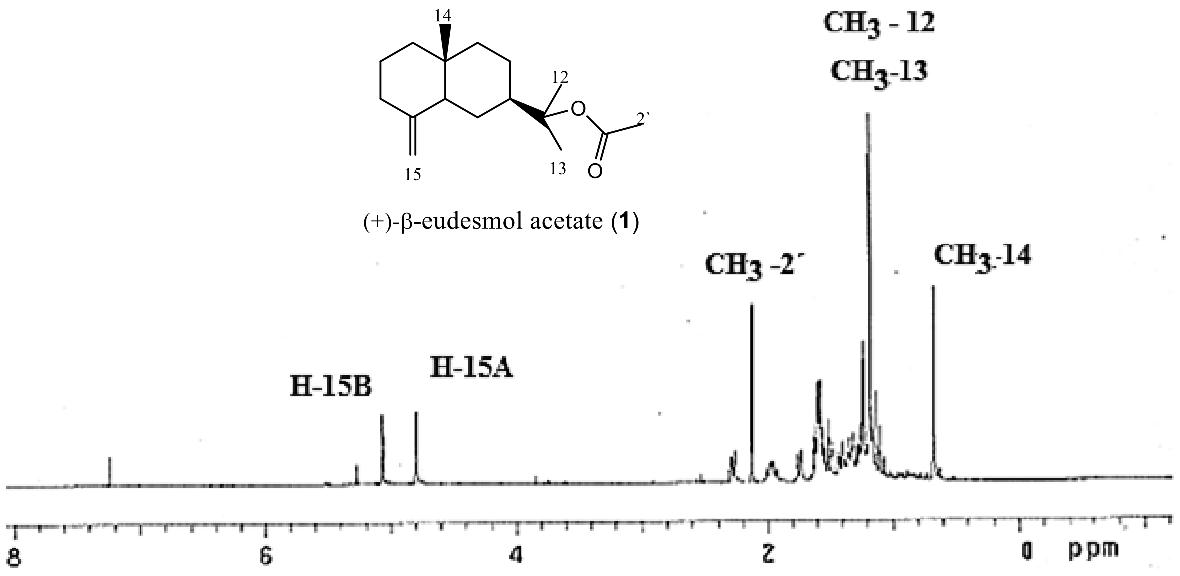

Figure S7. ^{1}H NMR spectrum (400 MHz; $CDCl_3$) of compound **1.**

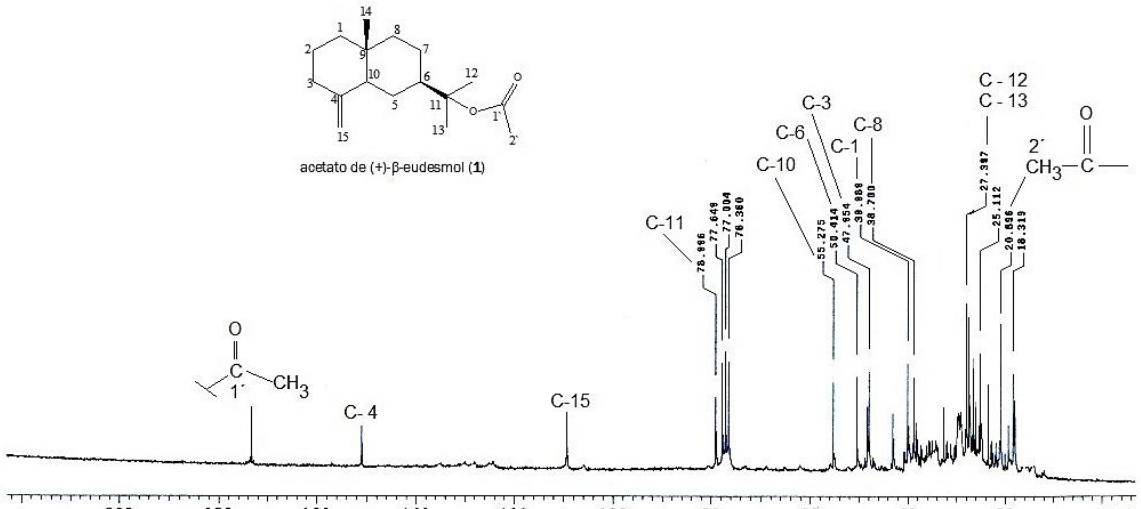

Figure S8. ^{13}C NMR spectrum (100 MHz, $CDCl_3$) of compound **1.**

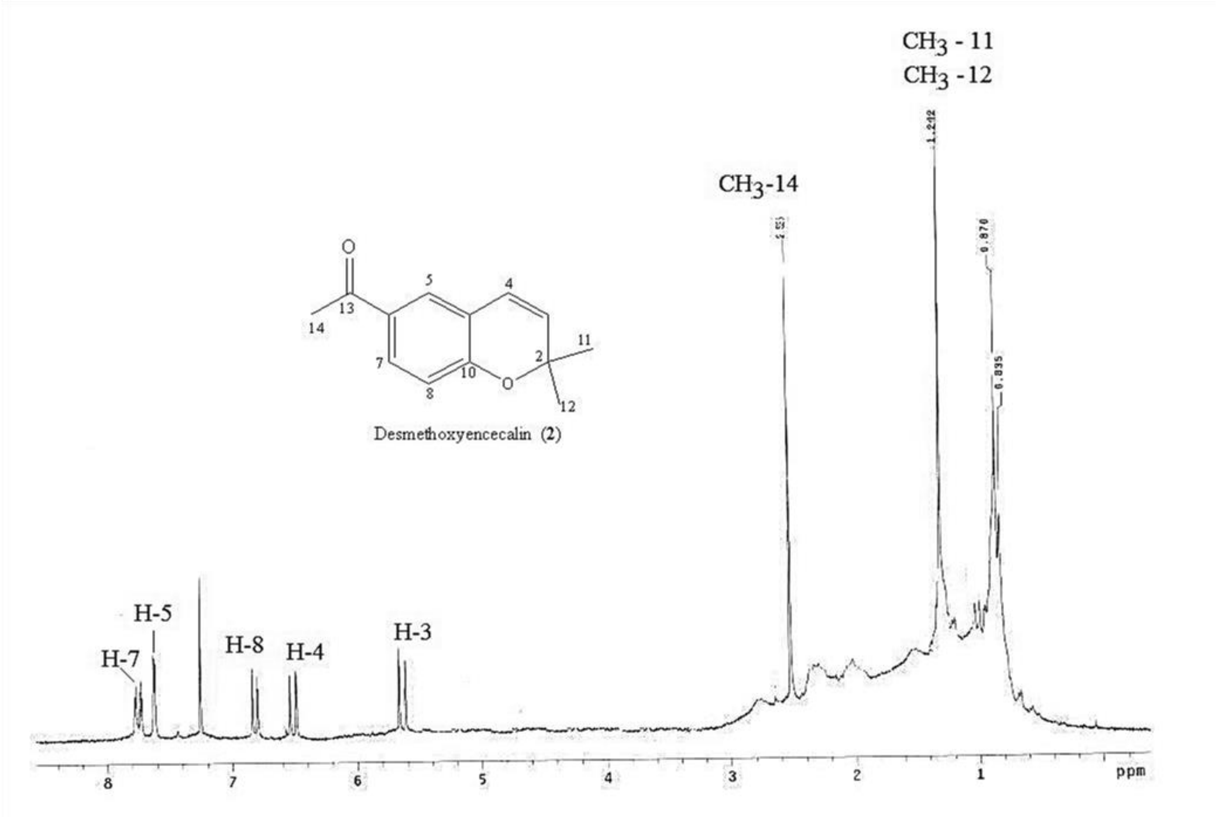

Figure S9. ^{1}H NMR spectrum (400 MHz; $CDCl_3$) of compound **2.**

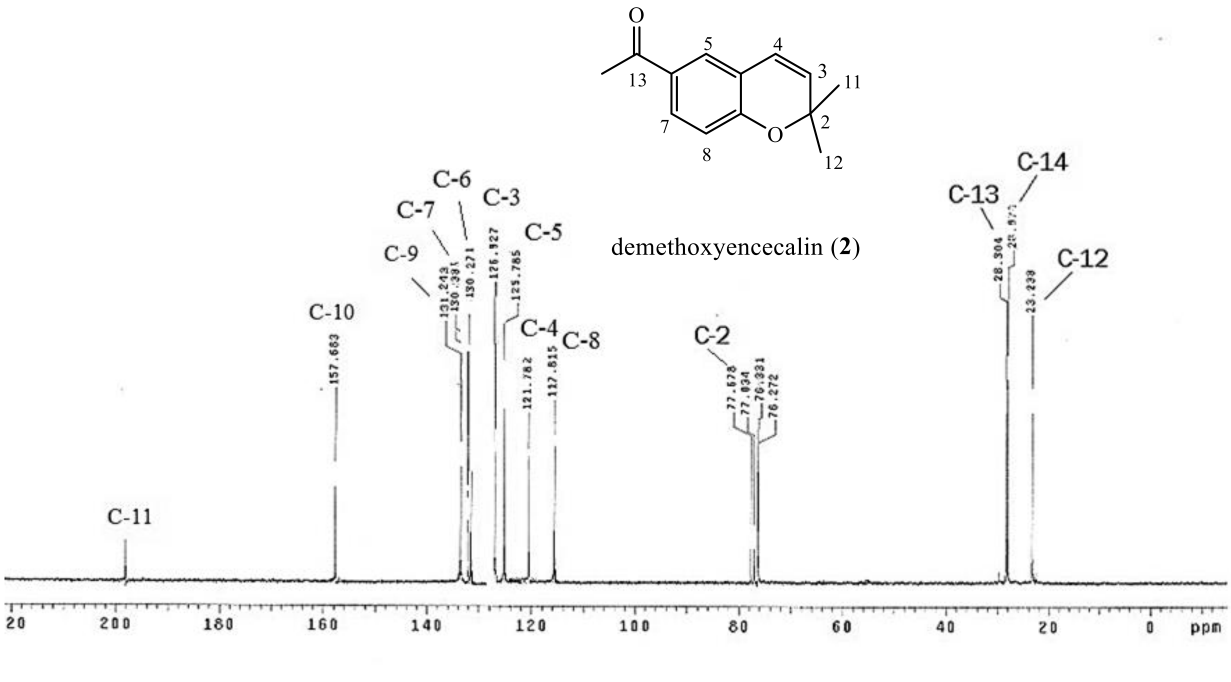

Figure S10. ^{13}C NMR spectrum (100 MHz, CDCl3) of compound **2.**

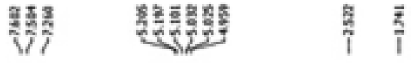

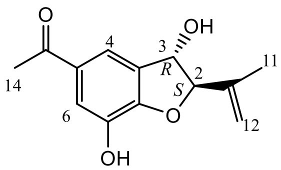

5-acetyl-7,3α-dihydroxy-2β-(1-isopropen)-2,3-dihydrobenzofuran (**5**)

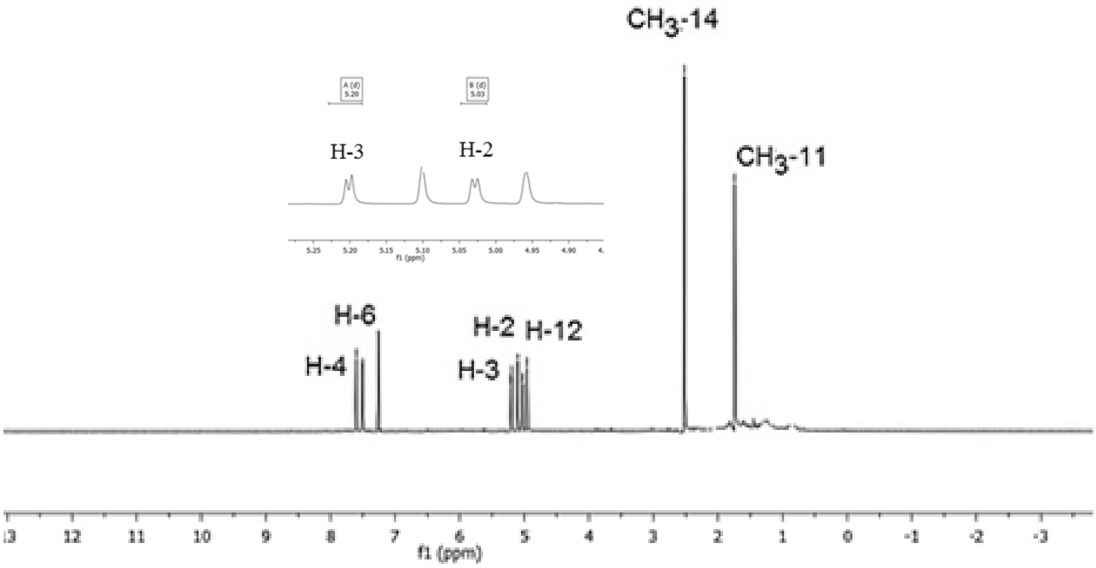

Figure S11. ^{1}H NMR spectrum (500 MHz; $CDCl_3$) of compound **5.**

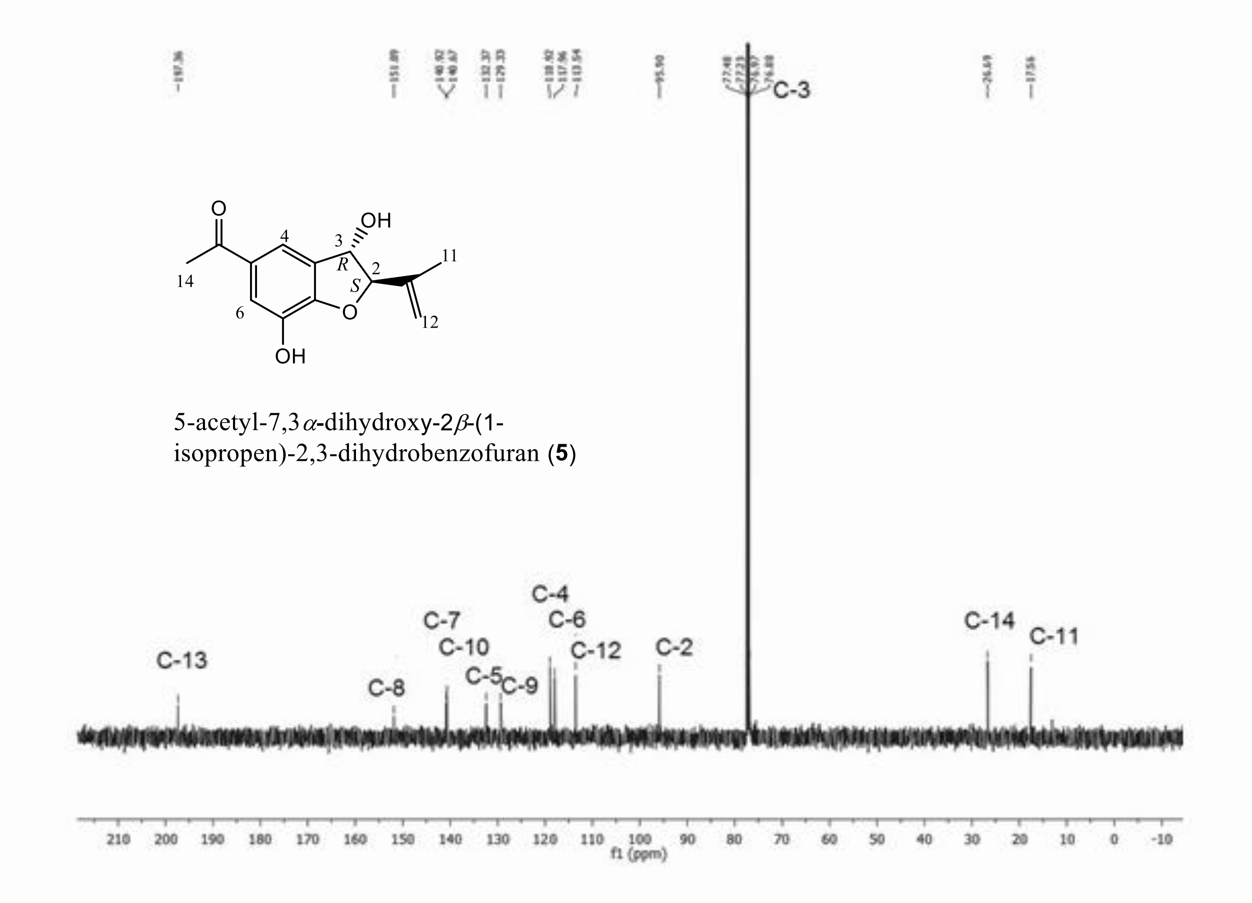

Figure S12. ^{13}C NMR spectrum (125 MHz, CDCl3) of compound **5.**

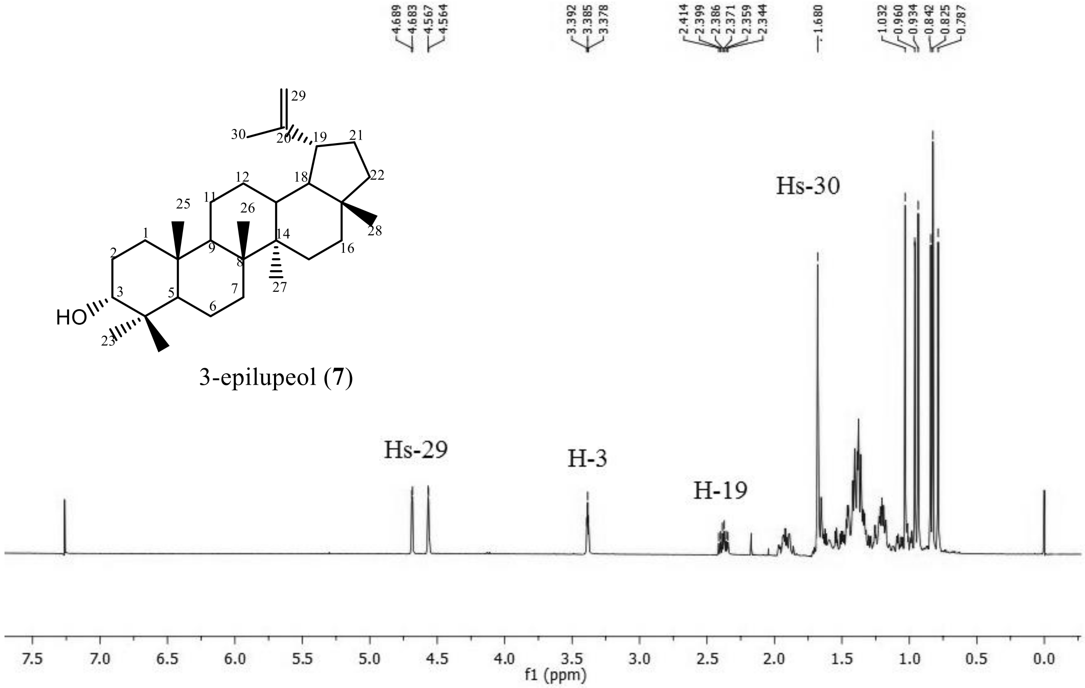

Figure S13. ^{1}H NMR spectrum (400 MHz; $CDCl_3$) of compound **7.**

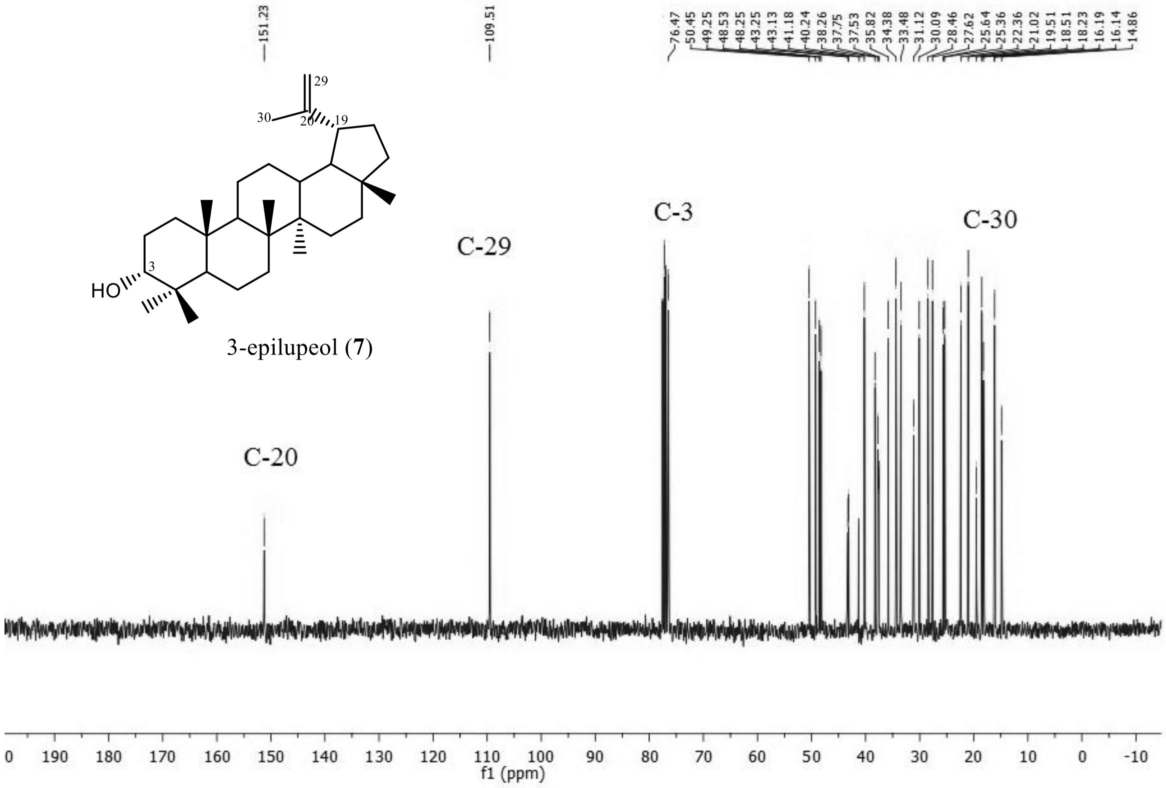

3-epilupeol (**7**)

Figure S14. ^{13}C NMR spectrum (100 MHz, $CDCl_3$) of compound **7.**
